# Resurgence of Polymyxin B for MDR/XDR Gram-Negative Infections: An Overview of Current Evidence

**DOI:** 10.1155/2017/3635609

**Published:** 2017-07-06

**Authors:** Suneel Kumar Garg, Omender Singh, Deven Juneja, Niraj Tyagi, Amandeep Singh Khurana, Amit Qamra, Salman Motlekar, Hanmant Barkate

**Affiliations:** ^1^Institute of Critical Care Medicine, Max Super Specialty Hospital, Saket, New Delhi 110017, India; ^2^Department of Critical Care and Emergency Medicine, Sir Ganga Ram Hospital, Rajinder Nagar, New Delhi 110060, India; ^3^Department of Medical Affairs, Wockhardt Ltd., Wockhardt Towers, Mumbai 400051, India

## Abstract

Polymyxin B has resurged in recent years as a last resort therapy for Gram-negative multidrug-resistant (MDR) and extremely drug resistant (XDR) infections. Understanding newer evidence on polymyxin B is necessary to guide clinical decision making. Here, we present a literature review of polymyxin B in Gram-negative infections with update on its pharmacology.

## 1. Introduction

Resistance to antibiotics is of significant concern to the health of the general population [1, 2]. Gram-negative bacteria (GNB) resistance is especially concerning to microbiologist and clinicians due to their rapid spread and limited treatment options [3]. Rapidly evolving antibiotic resistance to* Enterobacteriaceae *poses threat to existing antibiotics. Appearance of New Delhi metallo-beta-lactamase 1 (NDM-1) leading to carbapenem resistance suggests rapidly changing microbial environment [4]. This spreading antibiotic resistance is not matched by development of equally effective antibiotics [2]. This demands for effective utilization of old antibiotics that are possibly active against multidrug and extremely drug resistant (MDR and XDR) bacteria. Such felt need called for international collaborative efforts which was evident with ESCMID (European Society of Clinical Microbiology and Infectious Diseases) conference held at Vienna in 2014 on revival of old antibiotics [5, 6].

Polymyxins are one of the frontline antibiotics which have been revived in the past few years [6]. Resurgence of polymyxins in GNB infections especially the MDR* Pseudomonas aeruginosa, Acinetobacter baumannii,* and* Klebsiella pneumoniae *has been significant [7]. Growing use of polymyxins in GNB infections was identified and perceived in consensus for optimization on clinical use of polymyxins “*The Prato Polymyxin Consensus*” [8]. Given increasing use of polymyxin B in clinical settings, in this review, we provide current literature evidence of polymyxin B use as monotherapy and combination therapy for MDR and XDR Gram-negative infections.

## 2. Epidemiology of Resistance to Carbapenems

Carbapenem-resistant Enterobacteriaceae (CRE) has been reported with high mortality rates globally. CRE were unusual prior to 1992 but there has been significant rise worldwide [9]. A recent evaluation of CRE from the Unites States (US) reported incidence of 2.93 per 100,000 population. These bacteria were isolated most frequently from urine and had significant association with prior hospitalization [10]. Besides production of extended-spectrum beta-lactamase (ESBL),* Klebsiella pneumoniae* carbapenemase (KPC) is an essential component enabling resistance to most antibiotics including quinolones and aminoglycosides [9]. Recent observational reports from India suggest varied CRE prevalence between 12.26% and 71.25% [11–16]. Emergence of novel phenotypes like NDM-beta-lactamase has grown into major threats resulting in multidrug resistance. Recently in India, NMD-1 producing isolates majorly from* Escherichia coli* and* K. pneumoniae* were found to be resistant to all antibiotics except tigecycline and colistin [17]. Thus, resistance to carbapenems is a significant concern necessitating cautious use of existing antibiotics.

## 3. Polymyxins

Polymyxins which include polymyxin B and colistin (polymyxin E) are the “old” antibiotics which are used clinically. There has been a renewed interest in these antibiotics because of widespread resistance to newer antibiotics. Polymyxins are now labelled as “last resort” for MDR and/or XDR Gram-negative infections [20]. First approved for clinical use in 1940s, polymyxins were not favoured considering their toxicities. However, their growing use in critical care settings has helped understand in vitro and in vivo behaviour [21].

### 3.1. Chemistry

**Figure 1:**
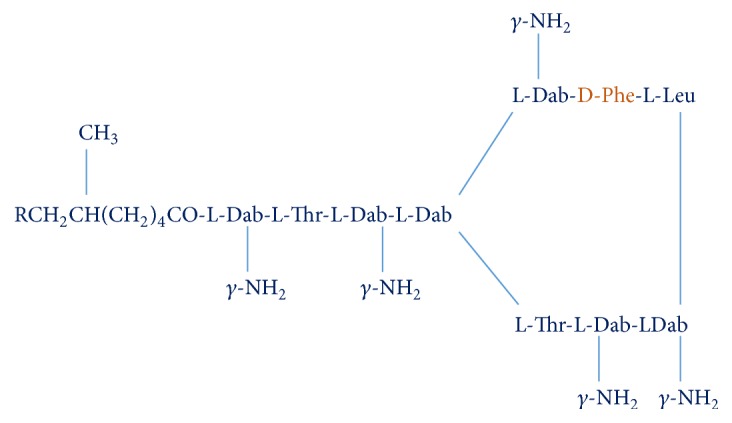
Structure of polymyxin B.

Polymyxins are composed of fatty acid chain (hydrophobic region) and amino acids (D and L) arranged in a cyclical heptapeptide ring. A tripeptide side chain binds cyclical ring to fatty acid chain. Single amino acid chain of D-leucine in polymyxin E is replaced by D-phenylalanine in polymyxin B (Figure 1) [20, 22].

Polymyxin B is commercially available as sulphate salt for parenteral administration [20]. Different polypeptides component in polymyxin B have different molecular formula. Polymyxin B component polypeptides include B1, B2, B3, and B1-I and sum of these constitutes minimum 80% for polymyxin B. Because of these components, there is batch to batch variation in commercial preparations [20].

### 3.2. Mechanism of Action

Antibacterial action of polymyxin can be segregated in two parts.* Firstly*, being positively charged, these cationic polypeptides interact electrostatically with lipopolysaccharide (LPS) present in outer cell membrane of GNB. These interactions displace positively charged Ca^++^ and Mg^++^ (stabilizers of lipopolysaccharide in outer cell membrane) leading to instability of cell membrane. A detergent-like effect leads to leakage of cell contents resulting in rapid bacterial death.* Secondly*, polymyxins are reported to have potent antiendotoxin activity and binding of polymyxins to lipid A component of LPS molecules neutralises it. However, the mechanism of septic shock prevention is unclear. It is believed that plasma endotoxin is immediately bound by LPS-binding protein, and the complex is quickly bound to cell-surface CD14 [7, 20].

### 3.3. Spectrum of Activity

Polymyxin B has bactericidal action against various Gram-negative bacteria including* Klebsiella *spp.*, Enterobacter *spp*., Pseudomonas aeruginosa, Acinetobacter *spp*. Escherichia coli, Salmonella *spp*., Shigella *spp.*, Citrobacter *spp*., Yersinia pseudotuberculosis, Haemophilus influenzae, Pasteurella *spp*., Bordetella pertussis, *and* Legionella pneumophila*. Majority of nosocomial pathogens are susceptible [20, 22].

Bacteria which are intrinsically resistant to polymyxins include Gram-negative isolates like* Burkholderia *spp*., Proteus *spp*., Providencia *spp*., Morganella morganii*, and* Serratia *spp. Additionally,* Brucella *spp.,* Neisseria *spp., and* Chromobacterium *spp. isolates are also resistant. All Gram-positive bacteria and anaerobes are also resistant to all polymyxins [20, 22].

Reported minimal inhibitory concentrations (MIC_50_ and MIC_90_) for major susceptible GNB were ≤1 and 2 mg/L for* Acinetobacter *spp., ≤1 and >8 mg/L for* Aeromonas *spp., ≤1 and 2 mg/L for* P. aeruginosa*, ≤1 and ≤1 mg/L for* E. coli*, and ≤1 and ≤1 mg/L for* Klebsiella* spp. respectively [21]. MIC_90_ for most isolates of* B. cepacia, S. maltophilia, Proteus *spp*., Proteus mirabilis, Serratia *spp*.,* and other enteric GNB was 8 mg/L or above suggesting the intrinsic resistance in these bacterial isolates [21].

### 3.4. Resistance Mechanisms

#### 3.4.1. Susceptibility Breakpoints

Susceptibility testing of polymyxins was revised in 2007 by Clinical and Laboratory Standards Institute (CLSI).  Table 1 summarizes breakpoints for* Pseudomonas aeruginosa*,* Acinetobacter,* and* Enterobacteriaceae* [21].

#### 3.4.2. Mechanisms

Majorly, the mechanisms of resistance involve alterations in initial interaction of polymyxins with LPS.* Intrinsically resistant* isolates of* Proteus mirabilis*,* Burkholderia cepacia,* and* Chromobacterium violaceum* have modification in lipid A component of LPS in outer cell membrane resulting in reduced binding of polymyxins. The major change observed in LPS is that the 4′-phosphate moiety of LPS is linked to 4-amino-4-deoxy-L-arabinopyranose making isolates resistant to polymyxins.* Acquired resistance* in* Salmonella *spp. and* E. coli* is associated with reduced susceptibility to polymyxins because of lipid A modification. Lipid A alteration with 4-amino-4-deoxy-L-arabinose (L-Ara4N) and/or phosphoethanolamine (PEtn) tends to decrease the negative charge of LPS which leads to reduced binding and increased resistance to polymyxins. Presence of capsule is identified to be a critical factor for driving resistance in* K. pneumoniae* [21, 22]. For some bacterial isolates, conditions of culture medium are identified to be responsible for resistance to polymyxins [23, 24].

### 3.5. Pharmacokinetics

Despite its clinical use, understanding of pharmacokinetics (PK) was limited for polymyxin B. In recent years, PK of polymyxin has been studied with enough details. Kwa et al. studied the PK of polymyxin B in MDR Gram-negative infections in adults ≥ 16 years without renal dysfunction. In a dose of 0.3 to 1 million units administered once or twice daily for mean duration of 7 days, mean volume of distribution (Vd) reported was 42.7 L with a half-life of 13.6 hours. Mean clearance was 2.4 L/h. This first attempt to describe PK of polymyxin B was limited by small number (*n* = 9) of subjects [25]. For long it is believed that the dosage of polymyxin B should be based on the renal function [7]. A recent investigation by Sandri et al. [26] provides substantial details of population PK of polymyxin B in critically ill patients. Twenty-four patients (above 20 years) who had varying creatinine clearance received polymyxin B in a dose of 0.45–3.38 mg/kg/day. They showed significantly low interindividual variability (coefficient of variation, 32.4%) in total body clearance when the dose was scaled by total body weight and not by total creatinine clearance. Clearance of polymyxin B did not show any correlation with creatinine clearance. Clearance was also unaffected in patients who were on renal replacement therapy suggesting that polymyxin B does not require dose modification in patients on renal replacement therapy. Large amount of filtered polymyxin B is reabsorbed from kidneys, which shows linear relationship with creatinine clearance suggesting higher reabsorption with declining renal function. Major PK findings from this study are summarized in Table 2.

Further this study highlighted dosing of polymyxin B. Based on MICs of causative organisms, a high dose regimen (3 mg/kg/d) is necessary for MIC ≤ 2 mg/L wherein a loading dose should be considered. For MIC ≤ 1 mg/L (less severe infection), a usual dose of up to 2.5 mg/kg/d would be appropriate. However, for higher MICs, dose greater than 3 mg/kg/d cannot be advised because of safety concerns [26].

In another small study of 8 patients, PK data on polymyxin B revealed a peak plasma concentration of 2.38 to 13.9 mg/L at the end of 1-hour intravenous (IV) infusion. Unchanged drug recovery in urine was 0.04%–0.86% of the dose. Further study highlighted that clearance of polymyxin B is independent of renal function and is eliminated majorly by nonrenal pathways [27].

### 3.6. Pharmacodynamics

Polymyxin B time-kill studies against isolates of* P. aeruginosa, K. pneumoniae,* and* A. baumannii* demonstrated concentration dependent killing [22]. The killing was followed by regrowth of the organisms. Such isolates were reported to have higher MICs for polymyxin B. In an in vitro study of* P. aeruginosa*, dosing interval representing that of 12 and 24 hours was associated with emergence of resistant isolates as compared to shorter interval dosing [28, 29].

Development of resistance or reduced susceptibility of such isolates might favour use of combination therapy with polymyxins. The use of combination therapy in in vitro studies has been reported to be associated with reduction in regrowth of isolates and reduction in polymyxin B resistance and bactericidal activity even at sub-MIC concentrations of polymyxins. However, the clinical evidence with combination therapy is limited [20, 22].

## 4. Efficacy of Polymyxin B: Monotherapy

As discussed previously, polymyxin B has different polypeptide components. Tam et al. assessed potency of these components in an in vitro study against three standard wild-type bacterial strains and three clinical MDR strains of* P. aeruginosa*,* A. baumannii*, and* K. pneumoniae*. Broth dilution method was used for MIC determination. No substantial differences were reported in potency against standard and MDR strains suggesting differences in molecular structure may not have difference in antibacterial activity [30]. In another study, Thamlikitkul et al. [31] studied polymyxin B activity against carbapenem-resistant* A. baumannii* (CRAB). In 217 strains of CRAB from different patients, MIC_50_ and MIC_90_ values were 0.5 and 1 mg/L, respectively. With a breakpoint of ≤2 mg/L, 98.2% strains were identified to be susceptible establishing efficacy of polymyxin B in CRAB infections. This finding can further be substantiated with findings from Mexico wherein Rosales-Reyes and colleagues [32] demonstrated 100% susceptibility of highly lethal (28.2% mortality) and biofilm producing clone (92.9% strains)—MDR* A. baumannii*—to polymyxin B. This clone was resistant to major antibiotics including aminoglycosides, cephems, carbapenems, and fluoroquinolones. These findings suggest superior efficacy of polymyxin B against MDR and biofilm producing* A. baumannii* isolates.

Polymyxin B is being considered as last resort in MDR Gram-negative infections. In a retrospective analysis of critically ill children (≤15 years) with MDR Gram-negative infections (*n* = 14), polymyxin B administered in a dose of 40,000 IU/kg/day resulted in survival of 57.1% children. Among bacterial isolates which included* Acinetobacter *spp*., P. aeruginosa, K. pneumoniae, *and* Enterobacter *spp., 100% sensitivity to polymyxin B was reported. Nephrotoxicity was evident in three cases [33]. Polymyxin B was observed as the modality to treat MDR Gram-negative infections. This calls for judicial use of this antibiotic in critical setting.

Kvitko et al. [34] retrospectively evaluated efficacy of IV polymyxin B (mean dose 141 ± 54 mg, twice daily) in comparison to other antibiotics in patients with* P. aeruginosa* bacteraemia. In 133 patients (33.8% with polymyxin B and 66.2% with others; most common being beta-lactams (83%)), in-hospital mortality was observed to be significantly higher (*p* ≤ 0.001) with polymyxin B (66.7%) than comparators (28.4%). Though mortality was higher with polymyxin B, optimized dosage utilization is crucial to reduce such outcomes. Preserving efficacy of polymyxin B to susceptible isolates is a priority undertaken to reduce and prevent emergence of resistance.

Nelson et al. [35] retrospectively studied efficacy of polymyxin B in bloodstream infections caused by carbapenem-resistant Gram-negative rods (*n* = 151,* K. pneumoniae* 60.9%,* A. baumannii* 21.2%, and* P. aeruginosa* 11.3%). Overall 30-day mortality was 37.8%. 63.6% were found to have clinical cure at day 7 of treatment. Post hoc analysis demonstrated a significantly higher mortality with dose <1.3 mg/kg/day (*p* = 0.02) but no difference in clinical cure at day 7 (*p* = 0.70). Acute kidney injury was observed to be significantly greater with dose of 250 mg/d and above (*p* = 0.03) which persisted in a multivariable analysis (odds ratio (OR) 4.32; *p* = 0.03). In another similar study, Elias et al. [36] explored impact of dose of polymyxin B on mortality outcome. In this retrospective evaluation, patients (*n* = 276) receiving polymyxin B for over 72 hours were included and subgroup analysis of microbiologically confirmed infections and those with bacteraemia was performed. Overall mortality rate was 60.5%. Septic shock (adjusted OR (aOR) 4.07), use of mechanical ventilation (aOR 3.14), Charlson comorbidity score (aOR 1.25), and age (aOR 1.02) were independent predictors of mortality. Polymyxin B in a dose of 200 mg/day and above was associated with significantly lower mortality outcome (aOR 0.43) and this effect was consistent in both the subgroups. But this dose had higher risk of severe renal impairment. These findings highlight the fact that higher dosage of polymyxin B benefits in terms of reducing in-hospital mortality. This association needs further exploration in a large, prospective, randomized trial. Increased risk of renal injury calls for careful look at coexisting factors that might contribute to renal damage. Obviating such confounding factors may prove beneficial in reducing severe renal injury with polymyxin B.

Dubrovskaya and colleagues [37] retrospectively evaluated the risk factors associated with polymyxin B monotherapy treatment failure in cases (*n* = 40) of carbapenem-resistant* K. pneumoniae* (CRKP). Clinical and microbiological cure were reported in 73% (*n* = 29/40) and 53% (*n* = 17/32) cases, respectively. Overall, 30-day mortality reported was 28%. After adjusting for septic shock, baseline renal insufficiency was found to be associated with 6 times greater chances of clinical failure. They also observed some of the breakthrough infections which were intrinsically resistant to polymyxin B. Thus, baseline renal dysfunction and subsequent development of resistant infections may lead to failure with monotherapy. Improvement of efficacy and preventing emergence of resistance in polymyxin B can be possible using it in combination with other antibiotics.

## 5. Efficacy of Polymyxin B: Combination Therapy

Combination therapy may prove beneficial in management of MDR and extremely drug resistant (XDR) organisms including superbugs. In this era of increasing bacterial resistance, combination therapy with polymyxin B holds promise in critical care setting. Being used commonly clinically, combination treatment holds the promise to effectively increase bactericidal activity and may reduce the development of resistance compared to monotherapy [38].

Rahim et al. [39] reported synergistic efficacy of polymyxin B and chloramphenicol in MDR NDM-producing* K. pneumoniae*. In these strains, chloramphenicol alone was ineffective whereas polymyxin B monotherapy was associated with rapid regrowth and emergence of resistance. With combination, there were no polymyxin resistant isolates. Scanning electron microscopy (SEM) features also were consistent with these findings. They found the formation of projections and blebs on the surface of bacterium which is consistent with mechanism of polymyxin B and they were denser with combination treatment. This provides insights that combination treatment may avert development of resistance to polymyxin B. This also adds to the finding that antibiotics considered “old” can be beneficial even in superbug infections when used in combination.

Carbapenem-resistant* A. baumannii *(CRAB) being a major nosocomial infection, combination treatments may prove beneficial. Lim et al. [40] evaluated three antibiotics—polymyxin B, rifampicin, and tigecycline alone and in combination in such infections. In 31 MDR isolates, all were susceptible to polymyxin B. In monotherapy time-kill studies, no antibiotic had bactericidal activity. In combination, polymyxin and rifampicin had highest bactericidal activity (41.9%) followed by polymyxin and tigecycline (29.0%) and tigecycline and rifampicin (22.6%).

Similarly, Hagihara et al. [41] reported that polymyxin B and tigecycline (200 mg) produced significantly greater reduction in bacterial density and the area under bacterial killing and regrowth curve (AUBC) compared to polymyxin B alone. Thus, combination therapy is an effective option for CRAB even in polymyxin B sensitive isolates.

Bowers et al. [42] assessed minocycline and polymyxin B combination in* A. baumannii* and reported that polymyxin B increased intracellular penetration and thereby concentration of minocycline as well as increased in vitro bactericidal activity. This further proves utility of combination treatment with polymyxin B.

In another study, Barth et al. [43] evaluated activity of polymyxin B in combination with imipenem, meropenem, or tigecycline in KPC-2 producing* Enterobacteriaceae*. Six strains including* K. pneumoniae *(*n* = 2),* Enterobacter cloacae *(*n* = 2), and* Serratia marcescens *(*n* = 2) had reduced susceptibility or resistance to polymyxin B and/or tigecycline and resistance to carbapenems. Polymyxin B plus carbapenem combination was most effective against* K. pneumoniae* and* Enterobacter cloacae *compared to tigecycline combination. For* Serratia marcescens*, polymyxin B plus meropenem was most effective combination providing synergistic bactericidal action.

**Figure 2:**
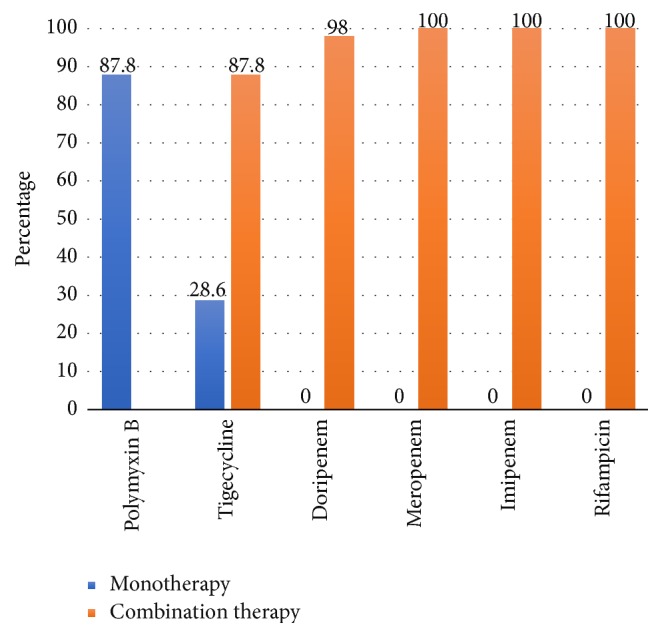
Bactericidal activity of all monotherapy and polymyxin B combination treatments in XDR* A. baumannii *[44].

In a study of extensively drug-resistant (XDR)* A. baumannii* (XDR-AB), Teo et al. [44] studied combination treatment of polymyxin B with imipenem, meropenem, doripenem, rifampicin, and tigecycline. Bactericidal activity of combination therapy was superior to monotherapy (Figure 2). This suggests combination therapy can be considered in suspected XDR infections.

**Figure 3:**
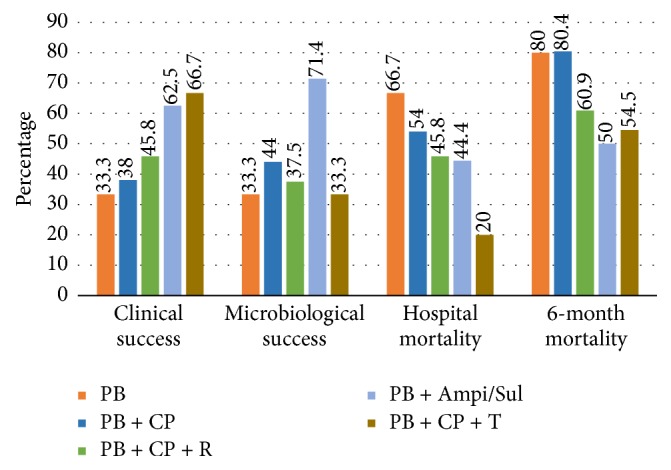
Comparison of polymyxin B combination treatments in different outcomes [45]. PB: polymyxin B, CP: carbapenem, R: Rifampin, Ampi/Sul: Ampicillin/Sulbactam, and T: Tigecycline.

Clinical studies on polymyxin B combination are limited. Data are available from small, retrospective, observational studies. Large, prospective, randomized trials are necessary to establish the benefits with optimal dosage selection [38]. In an observational cohort study, Crusio et al. [45] evaluated different polymyxin B combination therapies in carbapenem-resistant Gram-negative bacteria. Different infections included* A. baumannii* (*n* = 34/104),* K. pneumoniae *(*n* = 25/104),* P. aeruginosa *(*n* = 11/104), and multiple organisms (*n* = 34/105). Bacteraemia was present in 5 cases. Clinical success, microbiological success, hospital mortality, and 6-month mortality in 5 treatment groups are summarized in Figure 3. No significant differences were reported in all-cause hospital mortality as well as in 6-month mortality outcome. Age, severity of infection, and Charlson score had significant association with hospital mortality.

In XDR* A. baumannii* or* P. aeruginosa* infections, Rigatto et al. [46] reported significantly lower rate of 30-day mortality in combination treatment group compared to polymyxin B monotherapy (42.4% versus 67.6%, resp., *p* = 0.03). Even in multivariate analysis, the combination treatment was found to be independently associated with 30-day mortality. Particularly, combination was useful with beta-lactams or carbapenems in* A. baumannii* infections.* P. aeruginosa *associated mortality was significantly lower with combination as compared to monotherapy (*p* = 0.005). This provides a positive evidence for superior efficacy of polymyxin B based combination therapy in treating MDR and XDR Gram-negative infections. Further, use of a validated polymyxin combination therapy (based on combination selected after multiple combination bactericidal testing) was found superior to nonvalidated combination therapy and polymyxin monotherapy in reducing mortality in cases of XDR Gram-negative infections [47]. Testing bactericidal activity of combination agents and thereafter combining these agents can reduce infection related mortality. However, empiric combination should not be delayed in a critical setting. It may further be modified after sensitivity testing.

### 5.1. Clinical Safety

#### 5.1.1. Nephrotoxicity

Nephrotoxicity is a known adverse effect of polymyxins. Older studies and case reports suggested high incidence of nephrotoxicity but no objective definition of renal dysfunction was available and was referred mainly with intramuscular administration. Polymyxin B was reported to be associated with a higher incidence of renal toxicity compared to colistin/colistimethate sodium. But recent literature suggests lower nephrotoxicity rates even with polymyxin B. Increased membrane permeability leading to cell swelling due to influx of water and ions and resultant cell death is the suggested mechanism of renal injury due to polymyxin B. Fatty acid and D-amino acid component are considered to be responsible for cell injury. Nephrotoxicity due to polymyxin B is dose-dependent [48]. A brief review of recent studies of polymyxin B associated nephropathy is discussed below.

Ouderkirk et al. [49] reported 14% prevalence of ARF in patients treated with polymyxin B (*n* = 60). Those who developed ARF were older (mean age of 76 versus 59 years, *p* = 0.02). Higher mortality rate was reported (57% versus 15%, *p* < 0.02) in ARF cases. Similarly, Holloway et al. [50] reported ARF in 21.2% (*n* = 7/33) patients. None of the ARF required dialysis, and creatinine levels returned to normal range with discontinuation of polymyxin B in 71.4%  (*n* = 5/7) cases. Furtado et al. [51] reported nephrotoxicity in 9.4% of patients with* P. aeruginosa *associated nosocomial pneumonia treated with polymyxin B. Also, there was no difference in ARF occurrence in patients who had favourable or unfavourable outcomes.

Few studies tried to identify the factors associated with renal injury with polymyxin B. Bahlis et al. [52] in a retrospective cohort study identified 43% patients of renal injury by RIFLE (Risk, Injury, and Failure; Loss; and End-stage kidney disease) criteria. They observed hypotension (OR 2.79; *p* = 0.006) and concomitant vancomycin use (OR 2.79; *p* = 00.011) as independent predictors of renal injury. Similarly, Dubrovskaya et al. [53] in a retrospective cohort study evaluated 192 patients who received polymyxin B for over 72 hours. In a mean duration of 9.5 days of treatment, renal injury was found in 45.8% patients. They reported daily dose based on actual weight (hazard ratio (HR) 1.73, *p* = 0.022), concomitant vancomycin (HR 1.89, *p* = 0.005), and use of contrast media (HR 1.79, *p* = 0.009) as independent risk factors for nephrotoxicity. In another multicentre, retrospective cohort study, comparison of nephrotoxicity rates between colistimethate sodium (*n* = 121) and polymyxin B (*n* = 104) was performed by Phe et al. [54] to validate their findings of in vitro cytotoxicity study. Patients receiving polymyxin B for over 72 hours who had normal kidney function were assessed. In risk factors matched analysis, observed rates of nephrotoxicity were significantly (*p* = 0.004) higher with colistimethate sodium (55.3%) compared to polymyxin B (21.1%). On a multivariate analysis, significant and independent association of renal toxicity due to colistimethate was reported with age (OR: 1.04, 95% CI, 1.00, 1.07), treatment duration (OR: 1.08, 95% CI, 1.02, 1.15), and daily dose based on ideal body weight (OR: 1.40, 95% CI, 1.05, 1.88). A prospective comparison between two polymyxins is needed to further substantiate this finding.

A prospective cohort evaluation from Rigatto et al. [55] in 410 patients receiving polymyxin B for over 48 hours reported acute renal toxicity in 46.1% cases. Dose of polymyxin ≥ 150 mg/day was significantly associated with renal injury (HR 1.95, *p* = 0.01). Interestingly, the increased risk was maximal for dose range from 150 to 199 mg/day and no further significant increase was observed for even higher doses. They found renal injury as independent predictor of 30-day mortality (HR 1.35, *p* = 0.06) but the dose over 150 mg/day did not increase mortality. This paradox calls for careful patient evaluation. Higher dose may be associated with mortality but simultaneous renal injury is increased. Identifying underlying predisposing factors like hypotension, use of vancomycin, or any contrast media is essential. Correcting these abnormalities might help lower the incidence of renal injury with polymyxin B. In view of this, Rigatto et al. [56] studied mortality outcomes in patients of renal replacement therapy (RRT). In 88 RRT patients receiving polymyxin B (1.5 to 3 mg/kg/day) for over 48 hours, 30-day mortality was 51.1%. A daily dose above 200 mg was associated with lower mortality (HR 0.35, *p* = 0.03). Thus, a higher dose is effective in lowering mortality even in RRT cases.

#### 5.1.2. Neurotoxicity

Reported incidence of neuropathy with use of polymyxins is nearly 7%. Symptoms are like any other neuropathy and include weakness, paraesthesia, ophthalmoplegia, dysphagia, ataxia, and neuromuscular weakness sometimes leading to respiratory failure. Most of the neurotoxicity has been described for colistin/colistimethate sodium [48, 57]. However, no severe forms of neurotoxicity necessitating respiratory support have been reported in the last two decades [21]. Holloway et al. [50] reported one case of new-onset altered mental status and one with distal paraesthesia. Sobieszczyk et al. [58] reported neuropathy manifesting as seizures and neuromuscular weakness which were possibly related to polymyxin B in two (7%) cases. Weinstein et al. [59] recently reported two cases of polymyxin B induced neuropathy. First case was 60-year-old obese diabetic female with other multiple ailments and was on treatment with multiple medication including varenicline and quetiapine. Polymyxin B (loading dose 20000 U/Kg as two divided doses) was initiated for* K. pneumoniae *identified in urine culture which was sensitive only to polymyxin B. She developed oral paraesthesia within 1 hour of starting IV infusion. Second patient was 57-year-old male having ascending cholangitis. MDR* K. pneumoniae* susceptible only to polymyxin B, gentamicin, and trimethoprim-sulfamethoxazole was found in drain fluid culture. Multiple medications were introduced during hospitalization. For pancreatic abscess, patient was advised with 30-day treatment with polymyxin B and imipenem-cilastatin. After 30 days, oral and lower extremity paraesthesia were developed. Symptoms reversed with discontinuation of polymyxin B. There was no rechallenge attempted in either case. Though not commonly reported, caution is advised with increasing use of polymyxin B to monitor neurotoxicity.

#### 5.1.3. Congenital Anomalies

Though rare, risk of congenital anomalies exists for polymyxin B. Kazy et al. [60] reported crude OR of 0.8 for first trimester. Anomalies included cardiovascular malformations, neural tube defect, microcephaly, limb reduction defect, and congenital talipes equinovarus. Due to small number of cases, risk appears small though existent. Overall, there is limited data for polymyxin B and evaluation in larger sample is necessary to establish causal effect [21].

### 5.2. Tolerability of Polymyxin B

Overall, polymyxin B is well tolerated [58]. Milder adverse events may include rash, pruritus, dermatitis, and fever which are probably the result of histamine releasing action of polymyxin B [21].

### 5.3. Dosage and Administration of Polymyxin B

Currently, recommended dose of polymyxin B is 1.5 to 2.5 mg/kg/day administered intravenously in two divided doses as one-hour infusion. This dose is well tolerated in empirical setting [21]. Some reports suggest a dose of up to 3 mg/kg/day being used in clinical setting [26, 27]. Evidence suggests that daily dose of 200 mg and above is associated with better mortality outcomes. However, renal injury needs to be cautiously monitored at such higher dose. Doses above 3 mg/kg/day cannot be recommended due to safety concerns [26]. Evaluation of baseline renal function may be necessary but dosing is not affected by renal function as polymyxin B is majorly eliminated by nonrenal mechanisms. Prescribing polymyxin B in adequate dosage is essential to avoid underdosing in lieu of renal dysfunction [61, 62].

## 6. Place in Therapy

Polymyxin B has reemerged in clinical practice in recent years. Its use is likely to continue to increase since new drugs for the treatment of infections caused by MDR Gram-negative bacteria are beyond a distant horizon. Polymyxin B is a last resort therapy for MDR and XDR Gram-negative infections including those caused by* K. pneumoniae, A. baumannii, *and* P. aeruginosa*. Recent evaluations reporting PK data have made understanding of polymyxin B kinetics clear which is helpful in defining dosing regimens. Dosing based on actual body weight is helpful and should not be based on renal function. Efficacy against superbugs producing NDM-1 beta lactamases makes polymyxin B crucial in infection management. Intravenous administration has been effective in improving clinical, microbiological, and mortality outcomes not only in adults but in critically ill children also. Initial dose selection and titration are simple and more predictable for polymyxin B because of smaller interindividual variability and lack of impact of renal function on drug clearance. Therapeutic drug monitoring for polymyxin B lacks the significant difficulties that exist for colistin [63].

## 7. Conclusion

Recent studies of polymyxin B have provided additional understanding of pharmacokinetics with IV use and dosing based on weight with consideration of renal function and renal injury and factors associated with nephrotoxicity. However, there are still grey zones which need further evidence which include use in combination with other antibiotics and its comparison to monotherapy, mechanisms of resistance, pharmacokinetics in special patient groups like renal dysfunction, and measures to reduce nephrotoxicity. With polymyxin B being an essential antibiotic for MDR and XDR Gram-negative infections, polymyxin B antibiotic stewardship is strongly advised. Infection control and prevention measures should always supplement the antibiotic use.

## Figures and Tables

**Table 1:** Susceptibility breakpoints of polymyxins for major pathogens.

Organism	Profile		
	Susceptible	Intermediate	Resistant
CLSI recommendations [18]			
*P. aeruginosa*^*∗*^	MIC ≤ 2 mg/L	MIC = 4	MIC ≥ 8 mg/L
*Acinetobacter *spp.^*∗*^	MIC ≤ 2 mg/L	—	MIC ≥ 4 mg/L
*Non-Enterobacteriaceae*^*∗*^	MIC ≤ 2 mg/L	MIC = 4	MIC ≥ 8 mg/L
BSAC Recommendations [19]			
*Pseudomonas *spp.^*#*^	MIC ≤ 4 mg/L	—	MIC ≥ 8 mg/L
*Enterobacteriaceae *spp.^*#*^	MIC ≤ 4 mg/L	—	MIC ≥ 8 mg/L

^*∗*^For colistin and polymyxin B; ^#^for colistin only. MIC: minimum inhibitory concentration; CLSI: Clinical and Laboratory Standards Institute; BSAC: British Society for Antimicrobial Chemotherapy.

**Table 2:** Population pharmacokinetics of polymyxin B [26].

Parameter	Observation (range)
AUC_0–24_ (mg hour/L)	66.9 ± 21.6 (16.4–117)
*f*AUC_0–24_ (mg hour/L)	29.2 ± 12.0 (6.05–60.5)
C_ss,avg_ (mg/L)	2.79 ± 0.90 (0.68–4.88)
Renal Clearance (L/hour)	0.061 (0.018–0.377)
Urinary excretion (%)	4.04 (0.98–17.4)

AUC_0–24_: AUC over a day; C_ss,avg_: average steady-state plasma concentration; *f*AUC_0–24_: AUC for unbound fraction.
